# Rift Valley fever virus activates multiple cell death pathways in neurons

**DOI:** 10.1128/jvi.01742-25

**Published:** 2026-01-22

**Authors:** Kaleigh A. Connors, Zachary D. Frey, Matthew J. Demers, Morgan Midgett, Connor Williams, Douglas S. Reed, Zachary P. Wills, Amy L. Hartman

**Affiliations:** 1 Department of Infectious Disease and Microbiology, School of Public Health, University of Pittsburgh 6614, Pittsburgh, Pennsylvania, USA; 2 Center for Vaccine Research, University of Pittsburgh 6614, Pittsburgh, Pennsylvania, USA; 3 Department of Immunology, University of Pittsburgh 6614, Pittsburgh, Pennsylvania, USA; 4 Department of Neurobiology, University of Pittsburgh 6614, Pittsburgh, Pennsylvania, USA; St Jude Children's Research Hospital, Memphis, Tennessee, USA

**Keywords:** PANoptosis, cell death, Phenuiviridae, bunyavirus, neuron, viral encephalitis, Rift Valley fever

## Abstract

Neuronal injury and death contribute to long-term impairments and lethality during viral encephalitis. Rift Valley fever virus (RVFV), an arbovirus with epidemic potential, can manifest as late-onset encephalitis in humans, yet this disease outcome remains understudied. A lethal rodent model of RVF encephalitis is characterized by a dysregulated immune response, neuronal necrosis, and blood-brain barrier breakdown that precedes lethality. In this study, we built upon this prior work using both *in vivo* and *in vitro* models to interrogate the mechanism of cell death in neurons during RVFV infection. We found an increase in proteins associated with apoptosis, pyroptosis, and necroptosis in the brains of animals that succumb to lethal RVFV encephalitis. We then focused on identifying the primary cell death pathways in primary cortical neurons, which were highly susceptible to infection by pathogenic and attenuated viral strains. Using immunoblotting, immunocytochemistry, and in-cell western assays, we found that neurons infected with RVFV resulted in the activation of multiple cell death pathways, leading to neuron cell death. These findings further our understanding of the impact of RVFV infection, which is critical to identifying therapeutics that support neuron integrity and minimize injury during viral encephalitis.

**IMPORTANCE:**

Rift Valley fever may be accompanied by late-onset encephalitis in humans. Our lab has studied the *in vivo* mechanisms of neurological disease, yet the precise mechanisms of cell death in the central nervous system have been elusive. An understanding of the how and why of cell death from Rift Valley fever virus (RVFV) infection may guide the design of therapeutic interventions. Here, we use primary neurons to probe the mechanism of cell death following RVFV infection. We found that RVFV triggers multiple cell death pathways both in the brains of animals that succumb to lethal RVFV encephalitis as well as in *ex vivo* neuronal cultures. Induction of cell death occurs even with infection by an attenuated vaccine strain. These findings provide a platform for understanding cell death mechanisms caused by RVFV infection and identifying therapeutics that support neuron integrity during viral encephalitis.

## INTRODUCTION

Across Africa and the Arabian Peninsula, Rift Valley fever virus (RVFV) circulates in mosquitoes, with periodic epizootic disease in livestock and zoonotic spillover to humans resulting in public health and economic impacts (1). Livestock are infected via the bite of mosquitoes carrying RVFV, a *Phenuivirus* in the *Bunyaviricetes* class (2). Human infection typically occurs via mosquito bite or contact with infected fluids or tissue, although inhalation is also possible (3, 4). While most RVF cases are self-limiting, some individuals progress to more severe diseases, including late-onset encephalitis (5, 6). Incidence of severe disease varies greatly between outbreaks, with neurologic involvement reported in up to 17% of cases (4, 5, 7). Furthermore, neurologic involvement during RVF has been associated with a 53% case fatality rate (7).

Primary human clinical data on RVFV pathogenesis in the central nervous system (CNS) remain limited (8). Only a small number of human brain tissue samples obtained from autopsies during RVFV outbreaks have been studied (9). Some survivors of RVF encephalitis experience long-term sequelae, including both motor and cognitive disabilities, potentially resulting from neuronal death (5, 9, 10). Given that mature neurons have limited regenerative capacity following damage or disease (11), promoting the survival of neurons may be an approach to improving disease outcomes in humans. It is currently not known whether RVFV triggers one or more cell death pathways during infection of neurons, and this understanding may contribute to the design of better therapeutic approaches that support neuron survival during viral encephalitis.

**Fig 1:**
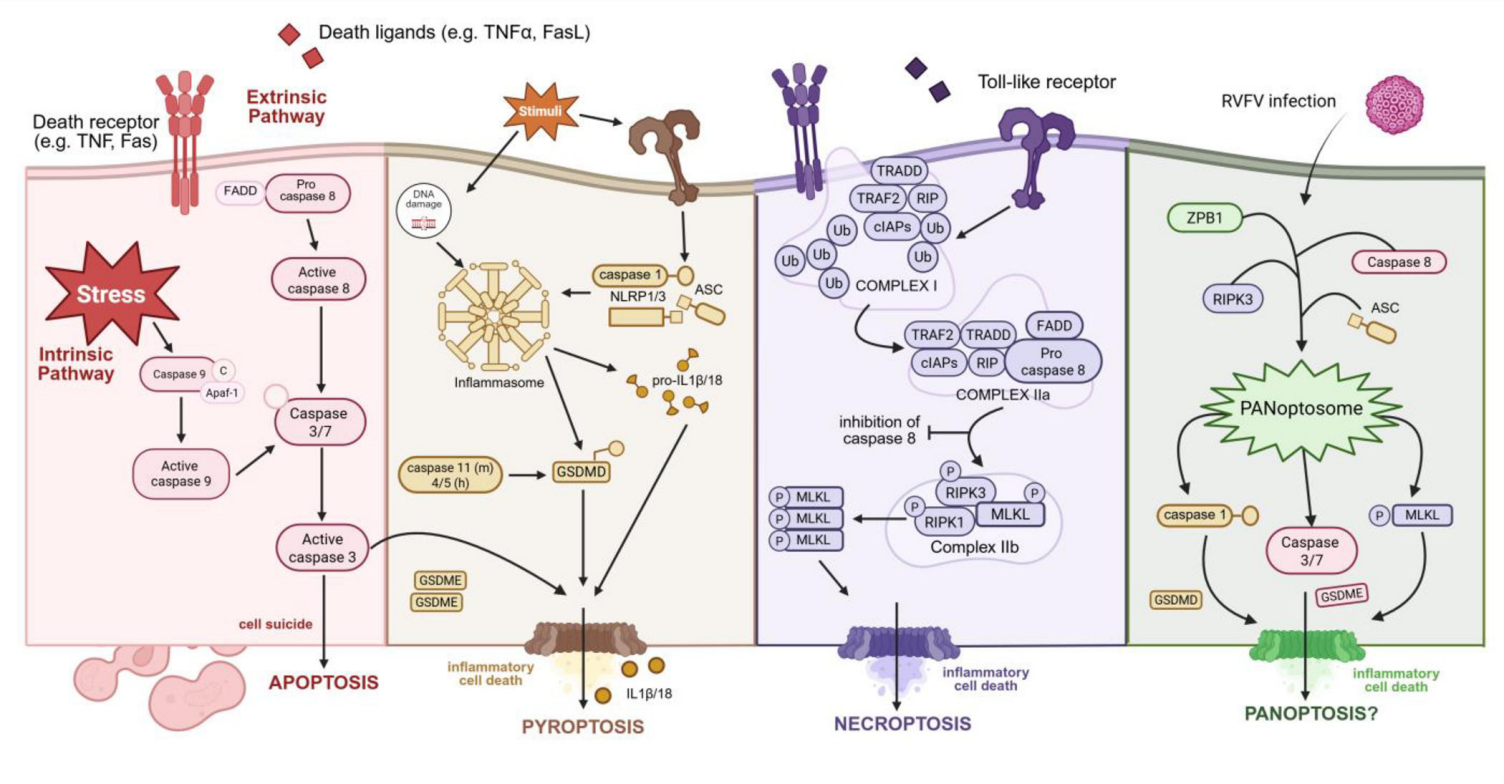
Key players in RCD pathways. Schematic representative of proteins involved in apoptosis, pyroptosis, necroptosis, and known mediators of PANoptosis. Apoptosis: activation of the caspase signaling cascade following extrinsic or intrinsic signaling through caspase 8 or caspase 9, respectively. These initiator caspases, in turn, activate caspase 3, leading to the dismantling of cellular components and programmed cell death. Pyroptosis: external or internal stimuli result in inflammasome assembly, which cleaves pro-caspase-1 into active caspase-1, enabling the cleavage of gasdermin D to form membrane pores, leading to cell swelling, lysis, and the release of pro-inflammatory cytokines. Additionally, pyroptosis may also occur through the activation of gasdermin E by caspase 3 cleavage, releasing the N-terminal pore-forming domain, resulting in membrane rupture. Necroptosis: through direct signaling from receptors or inhibition of caspase 8 activation, receptor-interacting protein kinases RIPK1 and RIPK3 are activated, which phosphorylate MLKL to form membrane-disrupting pores, leading to cell rupture and inflammation. PANoptosis: the PANoptosome formation follows stimulus, resulting in the activation of cell death mediators, including caspase-1, caspase-3, MLKL, gasdermin D, and gasdermin E. Created with biorender.com.

Viral infection in the CNS can trigger either direct pathogen-mediated effects, immune-mediated damage, or both, all of which can potentially lead to harmful neuroinflammation and neuronal cell death (12). Regulated cell death (RCD), a fundamental biological process responsible for tissue maintenance and homeostasis, functions as a key antiviral mechanism to halt viral replication, prevent further spread, and send a warning signal to neighboring cells (13, 14). Unlike many cell types and tissues, neurons have a limited ability to regenerate and tightly regulate cell death pathways; therefore, protecting them during viral infections is crucial to prevent permanent neurological deficits (11, 12, 15). Three RCD pathways—apoptosis, pyroptosis, and necroptosis—are well-described mechanisms of cell death and the focus of these studies (Fig. 1).

Apoptosis is non-inflammatory, crucial for development, and, as neurons mature, they raise the threshold at which apoptosis occurs (16, 17). In contrast, pyroptosis and necroptosis are highly pro-inflammatory processes (18–20). Pyroptosis involves cell swelling, membrane rupture, and the release of inflammatory molecules like IL-1β, while necroptosis is driven by the kinases RIPK1 and RIPK3 and is triggered by factors like TNF-α, contributing to inflammation and immune responses (18, 19). In 2019, the interaction between apoptosis, pyroptosis, and necroptosis and the activation of multiple RCD proteins was termed PANoptosis (P, pyroptosis; A, apoptosis; N, necroptosis), which has been implicated in viral and bacterial infections, including influenza A virus and herpes simplex virus (Fig. 1) (21–23). RVFV infection has previously been associated with the activation of apoptosis and pyroptosis, but not necroptosis, in non-neuronal cell types (24–26). Given recent observations that the non-structural protein of RVFV NSs sequesters cleaved caspase 3 in the nucleus during infection, we hypothesized that multiple mechanisms of cell death in neurons may contribute to their demise (27, 28).

In this study, we found that markers associated with apoptosis, pyroptosis, and necroptosis were activated in brain tissue obtained from rats that succumbed to RVFV encephalitis. Then, to pinpoint the RCD mechanisms initiated following RVFV infection in neurons, we obtained primary rat cortical neurons and used multiple orthogonal experimental methods to interrogate cell death pathway activation. We determined that RVFV infection resulted in the induction of apoptosis, necroptosis, and pyroptosis-associated proteins. Thus, multiple cell death pathways are activated in neurons during RVFV infection, suggesting that PANoptosis may contribute to their loss during infection. These findings support the need for additional studies to better understand RVFV infection in the CNS and how to prevent neuronal loss during viral encephalitis.

## RESULTS

### Multiple mediators of cell death detected in the brains of lethally infected rats

In our previous work, Lewis rats exposed to pathogenic RVFV via aerosol succumbed to lethal neurologic disease in 6–10 days with a 50% lethal dose (LD_50_) of 120 plaque-forming units (pfu) (29–31). In this model, disruption of brain tissue architecture, activation of microglia, dysregulated inflammatory response, and leukocyte infiltration are found in the brain immediately preceding lethality (30–33).

**Fig 2:**
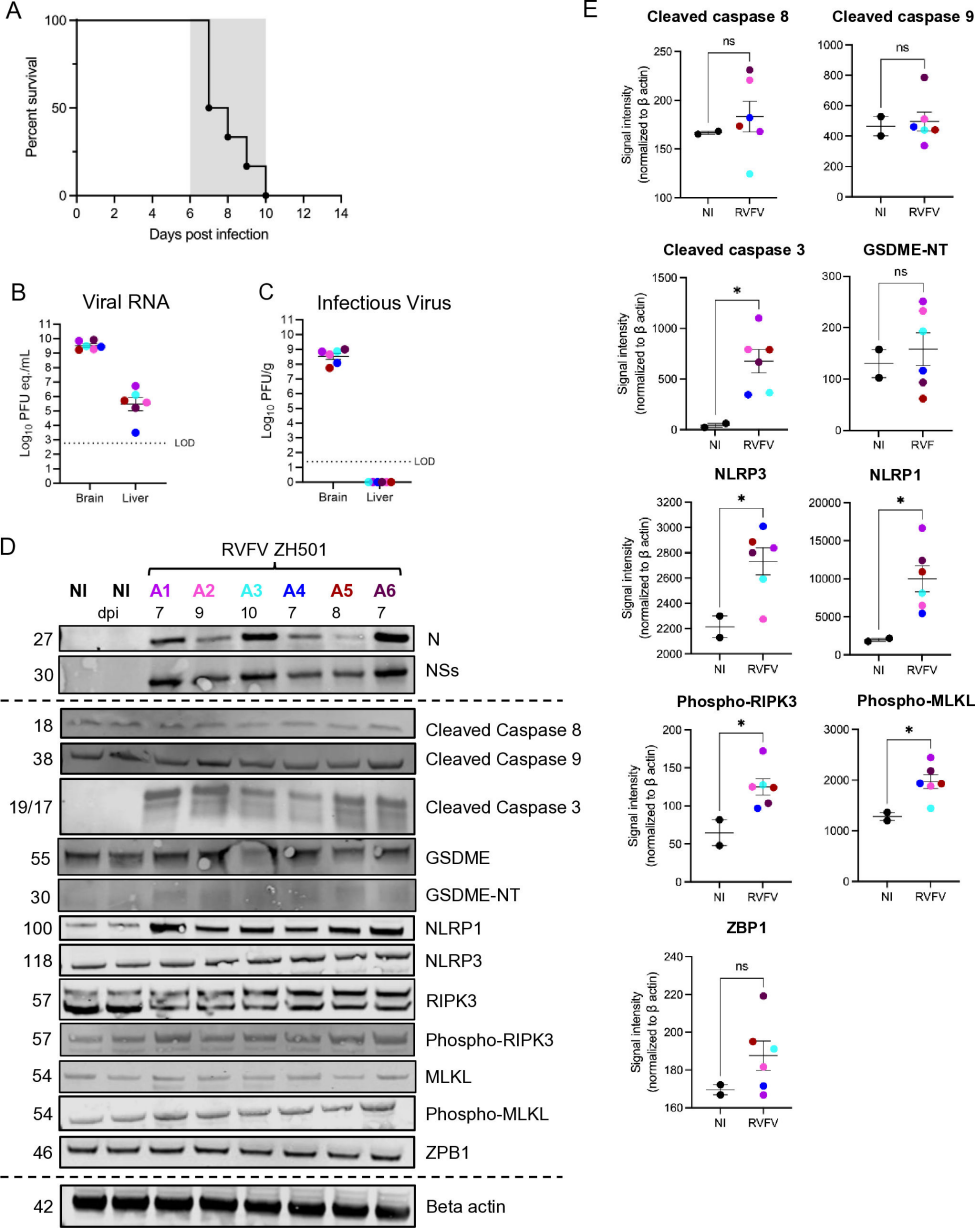
Rats infected with virulent RVFV by aerosol succumb to neurologic disease and express high levels of cell death-associated proteins in the brain at endpoint. (**A**) Survival of rats (*n* = 6) following inhalational exposure to RVFV strain ZH501 (average dose 230 pfu). Clinical window of neurologic disease displayed as a gray box between 6 and 10 dpi. (**B**) Viral RNA and (**C**) infectious virus were quantified in brain and liver tissue homogenate at endpoint. (**D**) Western blot of brain tissue homogenate probed with the indicated antibodies. The label above the lanes indicates non-infected controls (NI), infected animals, and dpi at which endpoint was met. (**E**) Quantification of western blot protein between uninfected and RVFV-infected rat brain tissue, measuring signal density normalized to beta actin. Colors in all panels correspond to matched samples from the same animal. Student’s *t* test was used to determine significance between groups, *P < 0.05; **P < 0.01; ***P < 0.001; ****P < 0.0001; and ns, no significance.

In the present study, rats were exposed by aerosol to an average of 230 pfu of the RVFV strain ZH501 (2 × LD_50_) and succumbed to lethal encephalitis 7–10 days post-infection (dpi) (Fig. 2A). Quantification of viral RNA in brain and liver tissue at endpoint (day of euthanasia due to severe neurologic disease) revealed high levels of viral RNA in the brain and liver, with titers reaching between 1 × 10^9^–10^10^ pfu equivalents/mL (pfu eq./mL) and 1 × 10^6^ pfu eq./mL, respectively (Fig. 2B). Infectious virus quantitated by viral plaque assay (VPA) at endpoint was detected up to 1 × 10^9^ pfu/g in brain tissue but not detected in liver tissue (Fig. 2C).

To initially evaluate changes in cell death markers in the brain, we measured the expression of RCD-associated proteins in brain homogenate obtained from rats at end-stage disease (Fig. 2D). We quantified the signal density of each band and found that cleaved caspase 3 (apoptosis), inflammasome markers NLRP1 and NLRP3 (pyroptosis), and both phosphorylated RIPK3 and phosphorylated MLKL (necroptosis) were significantly increased in brains obtained from RVFV-infected rats when compared with non-infected controls (Fig. 2E). In addition, we assessed the protein expression level of Z-DNA binding protein 1 (ZBP1), an innate immune sensor that recognizes cytosolic nucleic acids. ZBP1 has been implicated in the recognition of other RNA viruses and the formation of panoptosome complexes (22). In brains from rats infected with RVFV, we found elevated levels of ZBP1, though they were not significantly elevated compared to mock brains (Fig. 2E) (21, 22). Thus, in the context of whole brain homogenate obtained from animals at end-stage RVF disease, mediators of cell death from apoptosis, pyroptosis, and necroptosis were activated.

### Rift Valley fever virus replicates to high titer in primary neurons and results in loss of neuronal integrity

Given that both direct viral infection in the brain and the antiviral immune response to infection likely contribute to neuronal damage, we wanted to determine the direct impact of RVFV infection on neurons and thus used primary rat cortical neurons obtained from developing rat embryos. Primary neurons provide a physiologically relevant model, in terms of morphology, gene expression, and function, in which to study viral infection *in vitro*. RVFV encodes two non-structural proteins, NSs and NSm, both of which play contributing roles in immune evasion and virulence (24, 34–37). We used a recombinant ZH501 virus containing deletion of both of these nonstructural proteins (RVFV-delNSs-delNsm; herein “RVFV-delNSs/NSm”) (38) as a comparator against wild-type (WT) RVFV in these studies, given that both proteins have been previously implicated in cell death (24, 25, 28).

**Fig 3:**
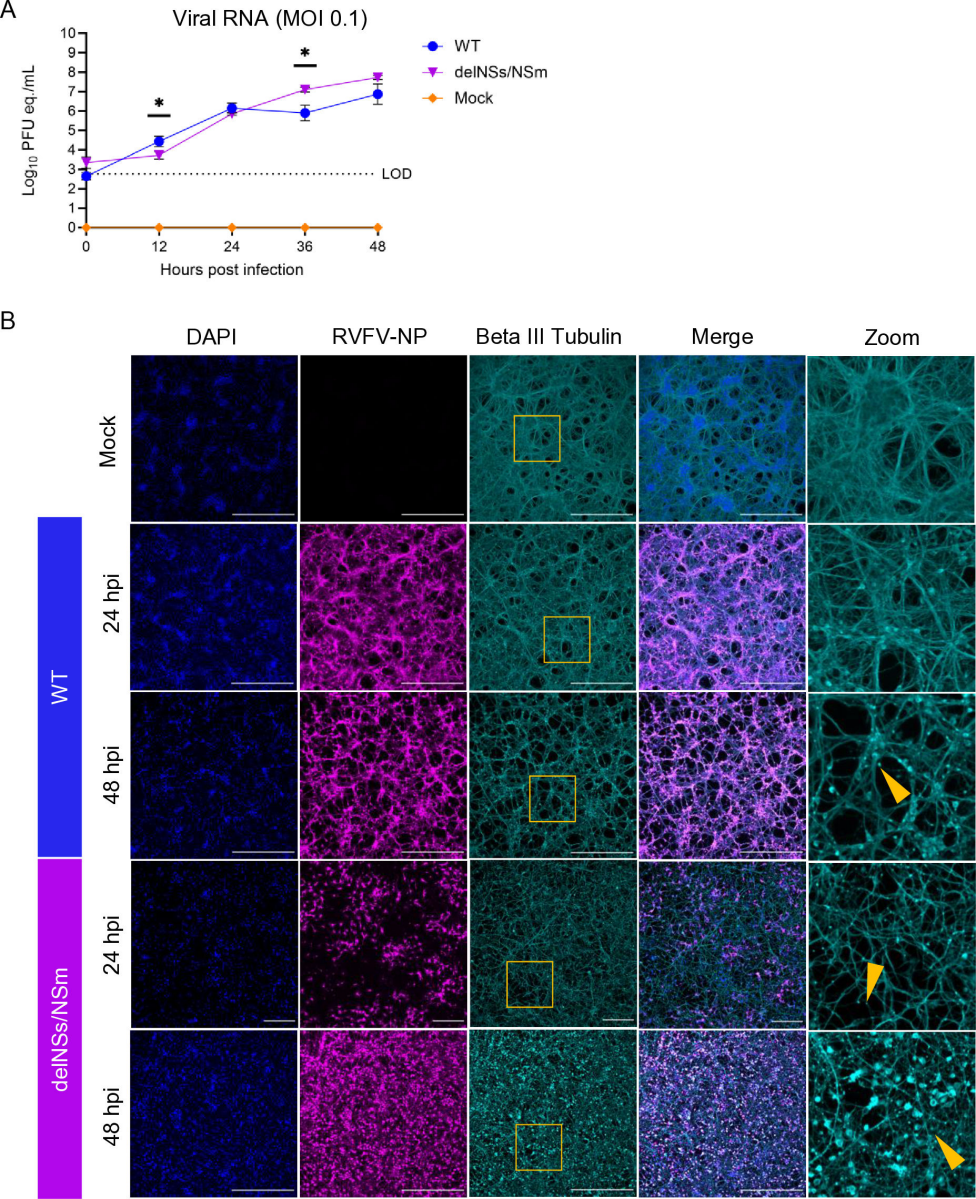
WT and attenuated RVFV strains replicate to high titers in primary cortical neurons. (**A**) Primary rat cortical neurons infected at an MOI of 0.1 with WT, RVFV-delNSs/NSm, or mock infected were sampled every 12 h. Viral RNA was collected from the supernatant and quantified using RT-qPCR. (**B**) Immunofluorescent microscopy of neurons infected with WT RVFV at an MOI of 0.1 at 24 and 48 hpi stained with anti-RVFV nucleoprotein (magenta), beta III tubulin (cyan), and counterstained with DAPI (blue). Zoom is included on the far right of beta III tubulin staining, with yellow arrows highlighting structural loss and punctate staining patterns. Yellow squares in the third panel are the sections magnified in the zoom. Magnification 60×, scale bar = 25 μm.

Primary rat cortical neurons were infected with an MOI of 0.1 of WT (strain ZH501) or RVFV-delNSs/NSm strains. Supernatant was collected every 12 h, from which viral replication was measured by RT-qPCR. Replication titers reached 1 × 10^5^ to 1 × 10^7^ pfu eq./mL by 48 h in neurons exposed to WT or RVFV-delNSs/NSm, respectively (Fig. 3A). The RVFV-delNSs/NSm strain displayed significantly decreased replication compared with WT at 12 h post-infection (hpi); however, RVFV-delNSs/NSm had a significantly higher viral titer at 36 hpi and was still increased over the WT virus at 48 hpi. Immunofluorescent microscopy illustrates widespread infection throughout the culture by 24 hpi in WT RVFV cultures, with progressive structural damage of neuronal processes between 24 and 48 hpi demonstrated by punctate staining and loss of dendritic structures (Fig. 3B, right panels, zoom). Fewer RVFV-positive neurons were observed in cultures infected with RVFV-delNSs/NSm at 24 h, and antigen staining increased through 48 hpi alongside neuronal integrity loss (Fig. 3B). These results demonstrate that rat neurons are permissive to direct infection with WT and RVFV-delNSs/NSm, with slightly delayed kinetics by RVFV-delNSs/NSm, which recovers over time. Both strains resulted in progressive neuronal structural loss between 24 and 48 hpi.

### Rift Valley fever virus infection of primary neurons results in neuronal cell death and activation of multiple RCD pathways

**Fig 4:**
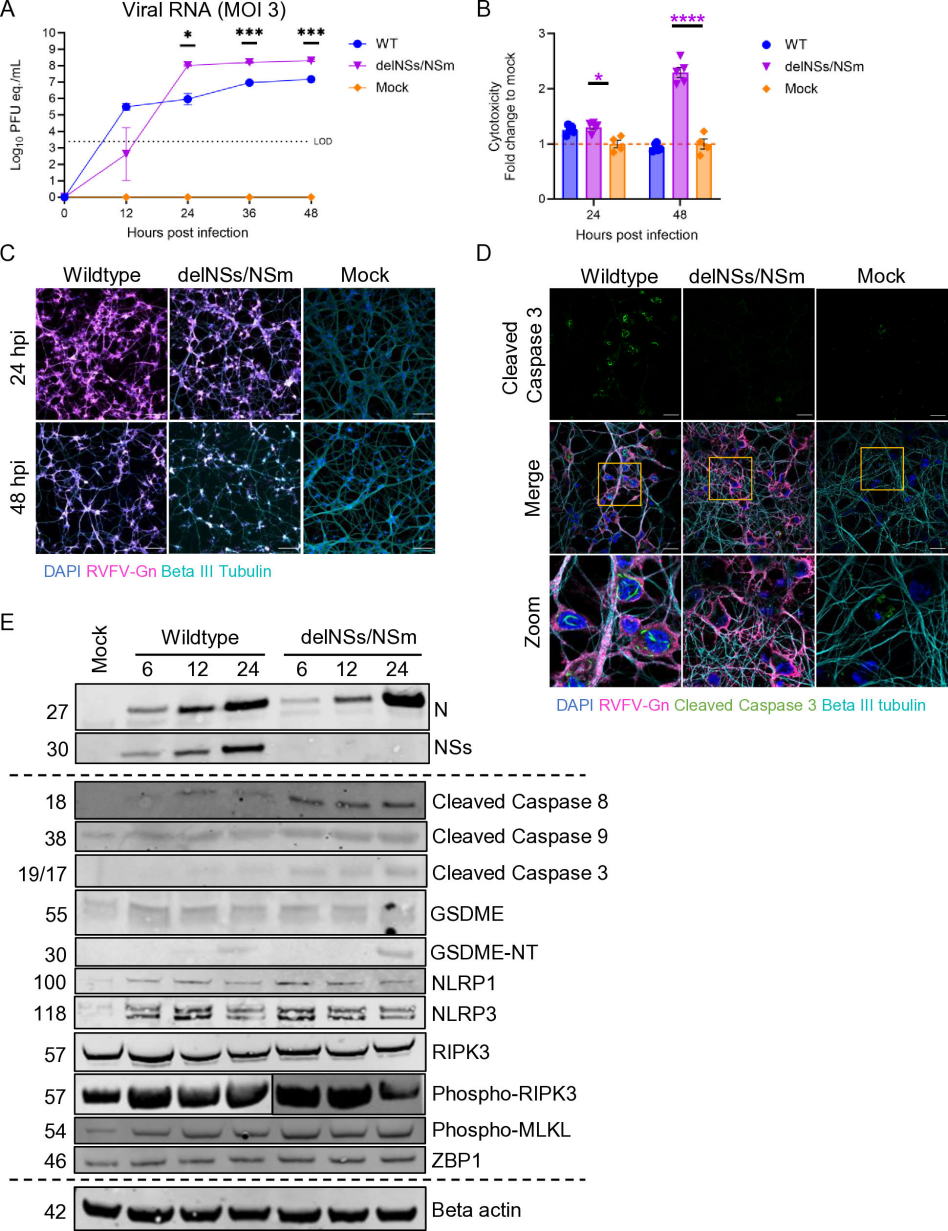
Primary cortical neurons infected with RVFV demonstrate activation of multiple RCD pathways. (**A**) Primary rat cortical neurons infected at an MOI of 3 with WT, RVFV-delNSs/NSm, or mock infected were sampled every 12 h. Supernatants were collected from each well, and viral RNA was quantified using RT-qPCR. (**B**) Cytotoxicity in primary rat cortical neurons following infection with WT and attenuated RVFV strains at 24 and 48 hpi quantified using the LDH-Glo Cytotoxicity assay. (**C**) Immunofluorescent microscopy of neurons infected with high MOI of WT, RVFV-delNSs/NSm, or mock-infected, fixed at 24 and 48 hpi, and stained with anti-RVFV-Gn (magenta), beta III tubulin (cyan), and counterstained with DAPI (blue). Magnification 20×, scale bar = 100 μm. (**D**) Neurons stained with anti-RVFV-Gn, cleaved caspase 3, and beta III tubulin antibodies at 24 hpi. Zoom of anti-cleaved caspase 3 staining (bottom panel). (**E**) RVFV-infected or mock-infected neurons were subjected to western blotting. Samples obtained from 6, 12, and 24 hpi from WT or RVFV-delNSs/NSm-infected neurons and a mock-infected control. Two-way ANOVA; *P < 0.05; **P < 0.01; ***P < 0.001; ****P < 0.0001; and ns, no significance.

To determine which RCD pathway is activated in neurons during RVFV infection, we infected primary rat cortical neurons with a high dose (MOI = 3) of WT or RVFV-delNSs/NSm. Viral RNA titer from infected neurons increased to 1 × 10^7^ and 1 × 10^8^ pfu eq./mL, respectively (Fig. 4A). We again observed a delay in viral replication in neurons infected with RVFV-delNSs/NSm at 12 hpi, which recovered from 24 to 48 hpi, at which point viral titer in RVFV-delNSs/NSm-infected supernatants was significantly greater than WT.

As a proxy for neuron viability, we measured the release of lactate dehydrogenase (LDH) at 24 and 48 hpi. Neurons infected with RVFV-delNSs/NSm released a high level of LDH compared to mock-infected neurons by 24 hpi (Fig. 4B). Surprisingly, neurons infected with the WT strain of RVFV had limited increases in LDH at 24 hpi and no additional increase in LDH release by 48 hpi (Fig. 4B). By immunocytochemistry, we observed a loss of neuronal processes in both WT and RVFV-delNSs/NSm-infected neurons between 24 and 48 hpi (Fig. 4C).

It is well established that the NSs protein of RVFV acts as a multifaceted virulence factor and co-localizes with cleaved caspase 3 in the nuclei of RVFV-infected hepatocytes and astrocytes (27, 28). Indeed, as shown here in primary neurons at 24 hpi, cleaved caspase 3 formed filaments in the nucleus in neurons infected with WT RVFV, but not in neurons infected with RVFV-delNSs/NSm (Fig. 4D). We were able to detect nuclear, filamentous cleaved caspase 3 in the nucleus of WT RVFV-infected cells as early as 6 hpi (Fig. S1A). Similarly, using an anti-RVFV-NSs antibody, filaments were beginning to form in the nuclei of RVFV-infected neurons as early as 6 hpi (Fig. S1B, left panel, zoom); by 24 hpi, most of the NSs staining was filamentous and intra-nuclear (Fig. S1B, right panel, zoom). These findings suggest that cleavage of caspase 3 and localization to the nucleus in RVFV-infected neurons occurs as early as 6 hpi, and the timing and location of this suggest co-localization with NSs. For this reason, we compared RCD pathways in neurons infected with WT and RVFV-delNSs/NSm at early time points of 6, 12, and 24 hpi to assess the initial activation of RCDs in neurons following infection at a high MOI.

We next measured RCD proteins using western blot on lysates from neurons infected with an MOI of 3 of either WT or RVFV-delNSs/NSm at 6, 12, and 24 hpi compared to a mock-infected control lysate obtained at 24 hpi (Fig. 4E). Virus-associated nucleoprotein (N) was detected in neurons infected with WT or RVFV-delNSs/NSm across all three time points, while NSs protein was only detected in neurons infected with WT RVFV, as expected. No viral proteins were detectable in mock-infected neurons. Apoptosis-associated caspases were activated in the presence of RVFV infection, with cleaved caspase 8 appearing in WT-infected cells by 12 hpi, and RVFV-delNSs/NSm-infected cells as early as 6 hpi. Similarly, we see an increase in cleaved caspase 3 at 12 hpi in WT and 6 hpi in RVFV-delNSs/NSm-infected neurons. The pyroptosis-associated protein gasdermin E, which is activated by caspase 3, was detectable in all samples (55 kDa band) and found to be cleaved at 24 hpi in both WT- and RVFV-delNSs/NSm-infected neurons (30 kDa band). Pyroptosis-associated inflammasome proteins NLRP1 and NLRP3 increased following infection with both WT and RVFV-delNSs/NSm. Necroptosis-associated proteins RIPK3 and MLKL were phosphorylated in the RVFV-infected neurons as early as 6 hpi. In addition, we probed RVFV-infected neurons for ZBP1 and found the protein level of ZBP1 increased following infection in both WT and RVFV-delNSs/NSm-infected neurons. These findings suggest that proteins associated with multiple RCD pathways were activated as early as 6 hpi in cortical neurons following infection with WT or RVFV-delNSs/NSm.

### Quantitative in-cell western assay reveals activation of multiple cell death pathways in neurons during RVFV infection

**Fig 5:**
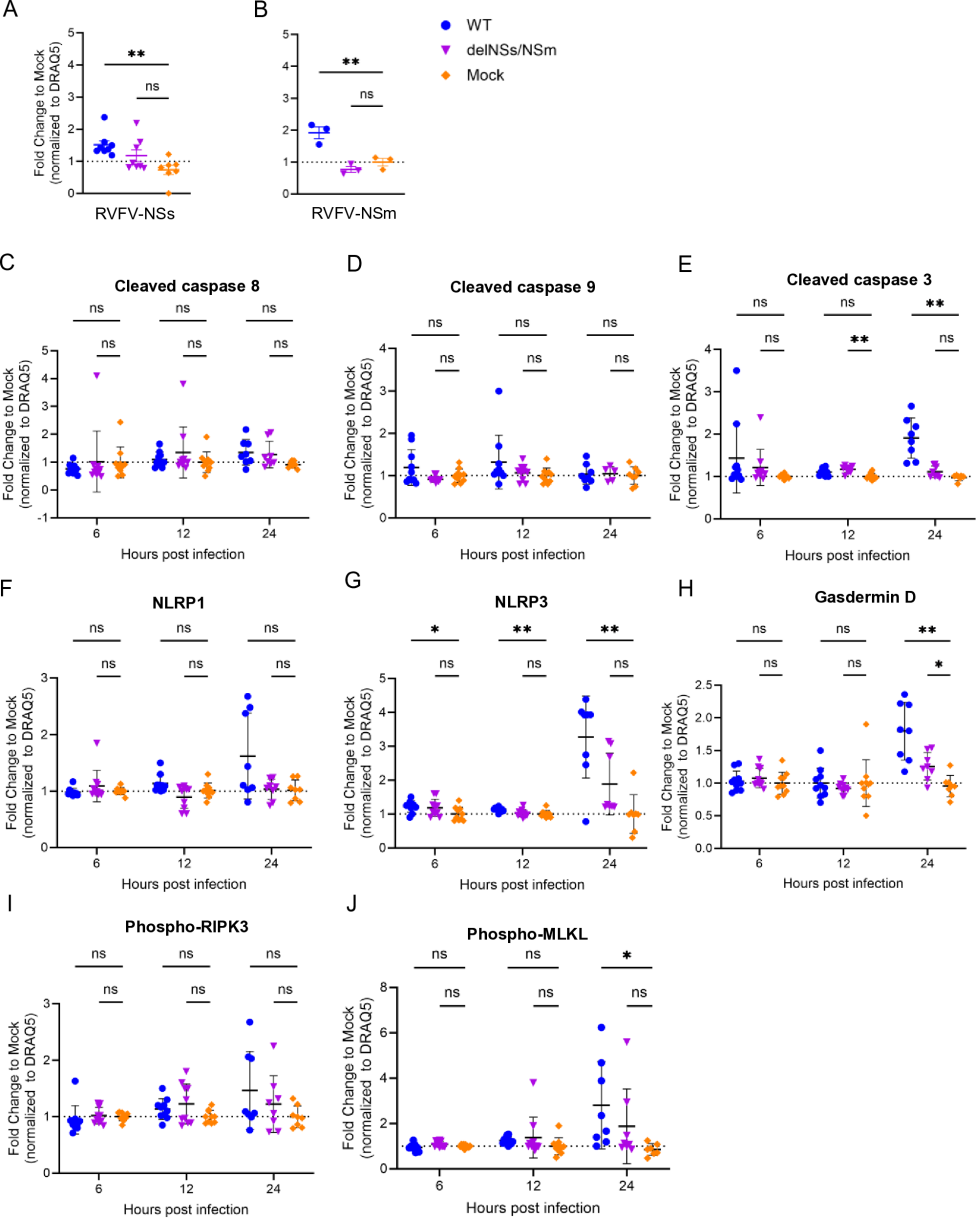
In-cell western assay shows activation of multiple proteins across RCD pathways during RVFV infection in neurons. Plates (96 well) were infected with WT or RVFV-delNSs/NSm at an MOI of 3, or mock infected. Whole plates were fixed with 4% PFA at 6, 12, and 24 hpi and then subjected to in-cell western assay. Plates were imaged using LICOR Odyssey, and the signal was quantified in each well using Image Studio software. The signal intensity of each well was measured and normalized to DRAQ5, then quantified as a fold change to mock-infected control wells. Neurons infected with WT or RVFV-delNSs/NSm were stained for anti-RVFV-NSs (**A**) or anti-RVFV-NSm (**B**). The fold change signal of WT- or RVFV-delNSs/NSm-infected neurons to mock was calculated for (**C**) cleaved caspase 9, (**D**) cleaved caspase 8, (**E**) cleaved caspase 3, (**F**) NLRP1, (**G**) NLRP3, (**H**) gasdermin D, (**I**) phospho-RIPK3, and (**J**) phospho-MLKL. Two-way ANOVA; *P < 0.05; **P < 0.01; ***P < 0.001; ****P < 0.0001; and ns, no significance.

To obtain a more granular understanding of which RCD pathways are activated in neurons following RVFV infection, in-cell western (ICW) assays were used (Fig. 5). ICW assay detects protein levels within intact, fixed cells using immunofluorescence, allowing for quantitative analysis while preserving cellular context, whereas traditional western blots involve lysing cells, separating proteins via gel electrophoresis, and detecting them on a membrane (39). Here, neurons were infected with WT or RVFV-delNSs/NSm, fixed at 6, 12, or 24 hpi, and then stained with antibodies to the indicated cell death proteins in addition to the far-red fluorescent nuclear dye DRAQ5 (40). The signal intensity of target proteins was normalized to DRAQ5, and RVFV-infected samples were compared to mock-infected controls and plotted as fold change over mock.

To assess the initial specificity of the ICW assay, we measured levels of viral proteins using anti-RVFV-NSs and anti-RVFV-NSm antibodies (Fig. 5A and B; Fig. S2A). Neurons infected with WT RVFV had significantly higher levels of NSs and NSm compared with mock-infected controls, while the level of signal from RVFV-delNSs/NSm-infected neurons, which lacks coding regions for both NSs and NSm, was not significantly increased over mock (Fig. 5A and B).

RVFV-infected neurons were compared to mock-infected neurons by ICW assay using antibodies targeting specific RCD proteins at 6, 12, or 24 hpi (Fig. 5). We did not detect a significant increase in cleaved caspases 8 or 9 following infection with WT or RVFV-delNSs/NSm by ICW assay, although we did observe a slight increase in cleaved caspase 8 at 24 hpi (Fig. 5C and D). Interestingly, cleaved caspase 3 was significantly increased in neurons following RVFV-delNSs/NSm infection at 12 hpi and WT RVFV infection at 24 hpi (Fig. 5E). Detection of the inflammasome marker NLRP1 was greater in WT-infected neurons by 24 hpi compared with mock-infected cells (Fig. 5F), while there were significant increases in NLRP3 in neurons following infection with WT RVFV as early as 6 hpi (Fig. 5G). We found that total gasdermin D levels increased in both WT- and RVFV-delNSs/NSm-infected neurons at 24 hpi (Fig. 5H). Phosphorylation of necroptosis marker RIPK3 was increased in WT- and RVFV-delNSs/NSm-infected neurons compared to mock by 24 hpi, while phosphorylated MLKL was significantly increased in WT-infected neurons at 24 hpi (Fig. 5I and J). Based on traditional western blot and ICW assays with quantification, RVFV infection of neurons induces activation of multiple cell death pathways.

### Caspase inhibition during RVFV infection reduces the accumulation of cleaved caspase 3 in neurons

**Fig 6:**
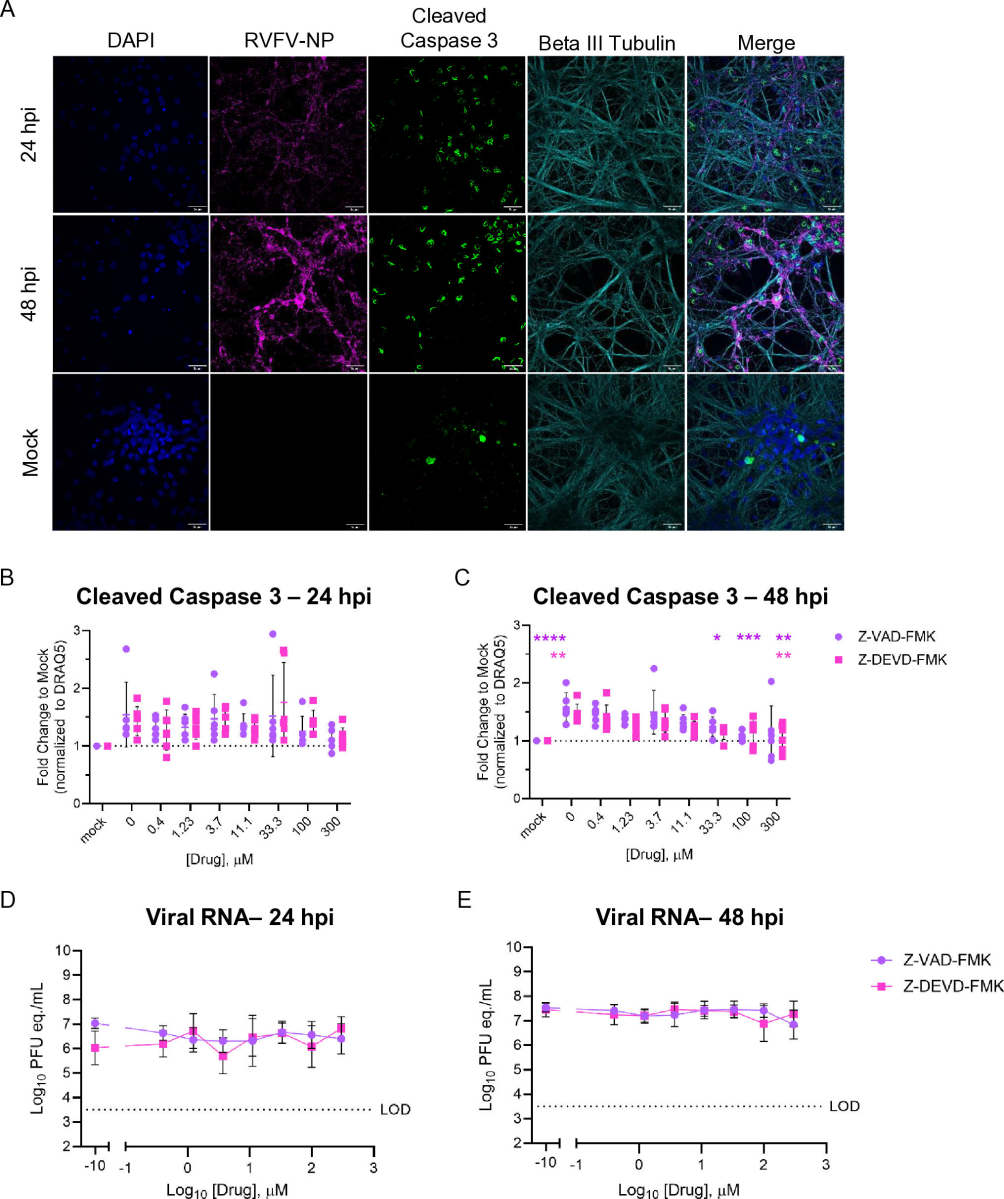
Caspase inhibition results in dose-dependent reduction of caspase 3 activation, but does not alter viral titers, in RVFV-infected neurons. (**A**) Primary cortical rat neurons were infected with an MOI of 0.1 WT RVFV or mock infected, and cover glass was obtained for imaging at 24 and 48 hpi. Neurons were stained with anti-RVFV-NP (magenta), anti-cleaved caspase 3 (green), anti-beta-III tubulin (cyan), and counterstained with DAPI. Magnification 60×, scale bar = 25 μm. (**B–E**) Neurons were pre-treated with Z-VAD-FMK or Z-DEVD-FMK at threefold dilutions in complete media 1 h prior to infection. Cells were infected with an MOI of 0.1 WT RVFV, then media were replaced with complete media containing Z-VAD-FMK or Z-DEVD-FMK for 24 or 48 hpi. (**B and C**) Expression of cleaved caspase 3 was quantified using ICW assay at 24 and 48 hpi. Signal intensity of cleaved caspase 3 was normalized to DRAQ5 and then compared to the expression of mock. (**D and E**) At 24 and 48 hpi, supernatants were collected to quantify viral RNA using RT-qPCR. Significance is represented by *P < 0.05; **P < 0.01; ***P < 0.001; ****P < 0.0001; and ns, no significance.

The reason for NSs colocalizing in the nucleus with cleaved caspase-3 is unknown. Viruses have evolved mechanisms to interfere with host defense mechanisms, including cell death, to promote viral replication and spread, which may explain the co-localization of RVFV-NSs and cleaved caspase 3 in the nuclei of RVFV-infected cells. Cleaved caspase 3 is readily detected intranuclearly in neurons infected with WT RVFV at an MOI of 0.1 at 24 and 48 hpi (Fig. 6A). To begin to understand how this mechanism impacts viral replication, we utilized two well-described caspase inhibitors, Z-VAD-FMK [benzyloxycarbonyl-Val-Ala-Asp(OMe)-fluoromethylketone] and Z-DEVD-FMK [benzyloxycarbonyl-Asp-Glu-Val-Asp(OMe)-fluoromethylketone].

Z-VAD-FMK irreversibly inhibits multiple caspases by covalently binding to their active-site cysteine residues, thereby blocking apoptosis. In contrast, Z-DEVD-FMK selectively inhibits caspase-3 and -7, key executioner caspases in the apoptotic pathway. Both inhibitors have been used to dissect caspase-dependent cell death mechanisms during viral infection (41–43). We hypothesized that in the presence of one of these caspase inhibitors, we would observe less caspase 3 activation in RVFV-infected neurons. If RVFV relies on the interaction between NSs and cleaved caspase 3 to facilitate infection, we would then expect a reduction in viral titers.

We estimated a 50% cellular cytotoxicity (CC_50_) for both compounds to be greater than 300 μM at both 24 and 48 h after treatment (Fig.S3). We then pre-treated neurons with threefold dilutions of Z-VAD-FMK or Z-DEVD-FMK for 1 h prior to infection with WT RVFV at an MOI of 0.1. Following adsorption, complete media containing compounds replaced the viral inoculum. Supernatants were collected at 24 and 48 hpi and used for the quantification of viral RNA titer, while 96-well plates were fixed and stained for cleaved caspase 3 for ICW analysis (Fig. 6B and C). Signal intensity of cleaved caspase 3 significantly decreased in a dose-dependent manner when Z-VAD-FMK- or Z-DEVD-FMK-treated neurons were compared with infected, untreated neurons at 48 hpi (Fig. 6C). However, the addition of caspase inhibitors did not significantly impact viral titers at 24 or 48 hpi (Fig. 6D and E). These experiments suggest that inhibition of cleaved caspase 3 in neurons does reduce the accumulation of activated caspase 3 in the cell but does not significantly impact viral replication *in vitro*.

## DISCUSSION

Viral infection in the CNS results in neuronal loss from direct infection and/or a dysregulated immune response, leading to long-term neurologic deficits in survivors or possible lethality. Neurologic symptoms of RVF can occur independently or concurrently with other severe forms of disease, such as hemorrhage or hepatic dysfunction (8). Treatment of patients with antiviral therapeutics may limit replication in the periphery, but most drugs have difficulty accessing the brain and achieving therapeutic levels (44, 45). Viral encephalitis, therefore, becomes difficult to treat. Currently, therapeutics that inhibit neuronal cell death are under investigation for neurodegenerative diseases, including Alzheimer’s, Parkinson’s, and amyotrophic lateral sclerosis, which may provide a tool for supporting neurons during viral infection (46–48). However, the mechanism by which RVFV induces neuronal cell death remains undescribed. Here, we characterize the mechanisms of RCD in neurons infected with WT and attenuated RVFV with the goal of providing potential targets for neuroprotective therapeutics.

We employed *in vivo* and *in vitro* methods to study the impact of RVFV on the brain, particularly in neurons. Previous studies show that animals with RVFV encephalitis display neurological symptoms, increased pro-inflammatory cytokines, and leukocyte infiltration before death (30–32). In this study, we observed elevated levels of apoptotic, pyroptotic, and necroptotic markers in the brain at endpoint disease, including significantly increased levels of cleaved caspase 3, similar to findings in infected mouse livers (27). Additionally, proteins linked to pyroptosis and necroptosis—NLRP1, NLRP3, phosphorylated-RIPK3, and phosphorylated-MLKL—were also elevated, indicating multiple cell death pathways are active during end-stage RVFV infection *in vivo*.

To better understand the direct impact of RVFV infection in neuronal cells, we utilized primary rat cortical neurons. In addition, we employed both a pathogenic and an attenuated RVFV strain to begin to assess the roles of virulence factors NSs and NSm in neuronal infection. NSs is the main virulence factor for RVFV that forms filaments in the nuclei of infected cells, which have been associated with inhibition of cellular machinery and processes to promote viral replication (34, 49–51). In addition, the formation of NSs filaments in the brain corresponds to amyloid-like structures and may play an important role in neurotoxicity (52). A functional NSs is required for neuroinvasion from the periphery, and its mutation results in disease attenuation (53, 54). Interestingly, we found that primary rat cortical neurons were highly susceptible to infection with both WT and attenuated strains of RVFV. Recent reports on RVFV infection in microglia and astrocytes demonstrate the overall susceptibility of resident brain cells to RVFV infection (28, 55). In addition, we found that neuronal infection with both WT and attenuated RVFV resulted in observable damage, as indicated by light microscopy and structural marker staining, thus suggesting that the NSs and NSm proteins may not be the primary drivers of cell death in neurons. The role of NSs and NSm in the neuropathogenesis of RVFV is an area in need of future study.

In line with recent findings, we detected cleaved caspase 3 in the nuclei of neurons infected with WT RVFV, but not with the NSm/NSs-deficient strain (28). By analyzing the timeline of caspase 3 activation in neurons, we found nuclear filaments beginning to form by 6 hpi, with RVFV NSs protein present in both cytoplasmic and nuclear regions at this time, consistent with prior reports (52). This corresponds to western blot data, whereby NSs protein is detectable in samples obtained 6 h after infection. In the current study, our data strongly suggest that cleaved caspase 3 and NSs colocalize in rat neurons, although we could not definitively demonstrate this due to antibody species incompatibility. Whether nuclear translocation of cleaved caspase 3 is dependent or independent of RVFV NSs remains unknown.

Using both western blotting and ICW assays, we detected RCD-associated proteins in denatured and native conformations. We readily detected cleaved caspases 8, 9, and 3 in cell lysates obtained from neurons infected with RVFV-delNSs/NSm, and to a lesser extent in WT-infected neurons. This is interesting considering how striking the cleaved caspase 3 filaments are visually by immunofluorescence assay in WT-infected cells, even early after infection. Furthermore, the activation of caspase 3 has been shown to increase along with RVFV replication (28). Using the ICW assay, we were able to quantify cleaved caspase 3 in WT-infected neurons and demonstrate the activation of apoptosis in the absence of substantial protein detected by immunoblot. Taken together, these results suggest that apoptosis is activated in neurons infected with both WT and attenuated RVFV. Importantly, this demonstrates how virus-host interactions may impact experimental readouts. It is proposed that pathogenic RVFV infection induces toxicity and the activation of apoptosis, which is subsequently delayed by nuclear sequestration of cleaved caspase 3 by NSs (28). This opens the door for redundant mechanisms of cell death to be induced during viral infection.

In addition to apoptotic markers, we observed increased levels of pyroptotic and necroptotic proteins, including NLRP1, NLRP3, gasdermin D, gasdermin E, phosphorylated-RIPK3, and phosphorylated-MLKL. Upon detecting microbial components, NOD-like receptors are intracellular sensors that trigger inflammatory responses via inflammasomes. Our group previously noted increased IL-1β levels in the brains of RVF-infected rats and in an *ex vivo* brain slice model (31, 56). Prior studies in immune cells confirmed NLRP3 inflammasome activation during RVFV infection (25, 26). Both NLRP1 and NLRP3 are expressed in the brain, with NLRP1 primarily in neurons and NLRP3 in microglia and astrocytes. We found elevated levels of both NLRP1 and NLRP3 in WT RVFV-infected neurons, while RVFV-delNSs/NSm induced a lesser increase in NLRP3. Activation of inflammasomes involves additional caspases, leading to the cleavage of gasdermin D, which forms pores in the cell membrane, resulting in cell lysis and the release of pro-inflammatory cytokines. Although we could not detect gasdermin D protein in rat brain or neuronal lysates, total gasdermin D levels were higher in RVFV-infected neurons (both WT and RVFV-delNSs/NSm) compared to uninfected neurons using the ICW assay. Recent findings indicate early gasdermin D cleavage in macrophages post-RVFV infection (26). Further supporting the activation of pyroptosis in neurons, we detected cleaved gasdermin E by western blot in both whole rat brains and primary neurons. Gasdermin E is a pore-forming protein from the gasdermin family that facilitates pyroptotic cell death following activation by cleaved caspase 3 (57). Together, our results support the activation of pyroptosis in neurons during RVFV infection.

We detected increased phosphorylated RIPK3 and MLKL levels in RVFV-infected brains and neurons by western blot and ICW assay. The stimulation of death receptors and pattern recognition receptors leads to necrosome activation of RIPK1 and RIPK3, which mediate necroptosis (15). Subsequent downstream signaling pathways, including the phosphorylation of MLKL by RIPK3, lead to membrane disruption and cell death. Alternatively, inhibition of caspase 8 activation can “trigger” necroptosis (58). A previous report found that RVFV-NSm inhibits apoptosis via caspases 8 and 9, slowing down cell death (24). We were able to detect cleaved caspase 8 in WT- and RVFV-delNSs/NSm-infected neurons, though we did not observe any significant differences in activation using ICW assay. The role of caspase 8 in the activation of necroptosis during RVFV infection requires further exploration. In addition, recent studies have demonstrated a neuron-specific, non-canonical function for RIPK3 in response to viral infection, and activation of RIPK3 during RVFV infection may play a protective role by recruiting immune cells to sites of infection (59–61). A related bunyavirus, SFTSV, can directly phosphorylate RIPK3 via the NSs protein and induce necroptotic cell death (62). Our findings suggest that RVFV induces RIPK3-MLKL necroptosis in neurons, the mechanism and function of which we plan to explore in future studies.

The findings of these studies suggest that in neurons, RVFV infection induces multiple cell death pathways involving lytic and inflammatory cell death mediated by pattern recognition receptors upon detecting pathogens or damage signals. Interestingly, while there were some differences in growth rate between WT and RVFV-delNSs/NSm viruses in our findings, both strains of RVFV induced substantial cell death by multiple pathways, indicating that neither NSs nor NSm is entirely responsible for the induction of or protection from cell death in neurons. We identified the activation of caspase 8, NLRP3, gasdermin D, RIPK3, and MLKL, which have been identified as components of panoptosomes. We investigated the role of ZBP1, a key mediator of IAV-induced PANoptosis, in activating PANoptosis during RVFV in neurons. However, we did not observe a significant increase in ZBP1 expression in rat brain homogenates or in primary rat neurons during RVFV infection. Other PANoptosome complexes have been described, including the absent in melanoma 2-panoptosome and pyrin-panoptosome (23). Still, it is likely other complexes mediating PANoptosis and other proteins involved in the execution of PANoptosis have yet to be described (23, 63). The involvement and composition of these complexes, which drive neuronal cell death during RVFV infection, are aims of future studies.

The purpose of the co-localization of cleaved caspase 3 and RVFV-NSs remains unknown. We hypothesized that the co-localization of RVFV-NSs with cleaved caspase 3 in the nuclei of infected cells may be an evolutionary mechanism that RVFV utilizes to prevent apoptotic cell death and promote viral replication. A similar mechanism whereby RVFV-NSs inhibits antiviral autophagy by sequestration of LC3 was recently demonstrated (51). Under this premise, we hypothesized that inhibiting caspase activation may reduce viral replication in neurons. Using two caspase inhibitors, Z-VAD-FMK and Z-DEVD-FMK, we demonstrate a reduction of cleaved caspase 3 in a dose-dependent manner at 48 hpi. However, this did not have a measurable effect on viral RNA titer at 24 or 48 hpi. This may be due to several factors, including the viral infection dose, timing of infection, or titer endpoints. Additional studies are needed to determine if inhibition of other proteins involved in neuronal cell death will limit viral replication and serve as potential neuroprotectant options.

A major limitation of this study was the availability of tools to use for immunoblotting and immunocytochemistry that were specific for rat tissue. We tested several antibodies targeting gasdermins (Table S1), many of which were undetectable by both traditional immunoblotting or immunocytochemistry techniques. In addition, there are few molecular and genetic tools available for rats, limiting our ability to perform critical *ex vivo* or *in vivo* studies using genetic knockouts. A key next step in better understanding the mechanisms of cell death in neurons following RVFV infection is the translation of these studies to both mice and humans. Our group and others have recently established and characterized novel immunocompetent mouse models of RVFV encephalitis (64, 65). Using these tools, alongside well-characterized genetic knockouts targeting cell death pathways, will be critical in establishing the importance of each pathway in neuronal cell death and the neuropathogenesis mechanisms of RVFV.

## MATERIALS AND METHODS

### Biosafety

Work with RVFV ZH501 was completed in biosafety level (BSL)-3 and animal biosafety level (ABSL)-3 conditions in the Division of Select Agents and Toxins-registered space within the Regional Biocontainment Laboratory (RBL) in the Center for Vaccine Research at the University of Pittsburgh. All RVFV tissue samples were inactivated using approved inactivation protocols before removal from BSL-3/ABSL-3 for further processing. Respiratory protection for laboratory workers was provided by 3M Versaflo Powered Air Purifying Respirators, and all excess infectious material was inactivated by immersion in Vesphene IIIse (Steris) diluted to 1:128 for at least 10 min.

### Animal work

All work with animals adhered to The Guide for the Care and Use of Laboratory Animals published by the NIH throughout the duration of the study. The University of Pittsburgh is fully accredited by the Association for Assessment and Accreditation of Laboratory Animal Care.

### Viruses

The ZH501 and RVFV-delNSs/NSm strains of RVFV used in these experiments were generously provided by Anita McElroy (University of Pittsburgh) and were derived from reverse genetics as described previously (66). Vero E6 (CRL-1586, American Type Culture Collection) cells were used to propagate each virus following standard cell-culture conditions in Dulbecco’s modified Eagle’s medium (DMEM) containing 2% or 10% FBS, 1% penicillin-streptomycin (pen/strep), and 1% L-glutamine. For quantitation, virus was measured using VPA (67) or 50% tissue culture infective dose (TCID_50_) methods and fully sequence confirmed (68).

### Rat experiment

Male and female Lewis (LEW/SsNHsd) rats were obtained from Envigo Laboratories (Indianapolis, IA, USA) between 8 and 10 weeks of age. Twenty-four hours prior to viral challenge, while under anesthesia, temperature chips were implanted subcutaneously in the dorsal area (Bio Medic Data Systems, Waterford, WI, USA). Rats were exposed to aerosols containing RVFV in a whole-body exposure chamber inside a class III biological safety cabinet located in the Aerobiology suite of the RBL as previously described (29, 69). Exposures were controlled by the Aero3G management platform (Biaera Technologies, Hagerstown, MD) (70). Total air into and out of the exposure chamber was set to 19.5 lpm to ensure one complete air change in the exposure chamber every 2 min. Aerosols were generated using a vibrating mesh nebulizer (Aerogen Solo; Aerogen Inc., Galway, Ireland) with airflow into the chamber at 7.5 liters per minute (Lpm). Secondary air (12.0 Lpm) included humidified air to achieve >80% relative humidity as previously described (71). An all-glass impinger (AGI; #7540, Ace Glass, Vineland, NJ, USA) with 10 mL of liquid media (DMEM containing glycerol and antifoam A), pulling vacuum at 6 Lpm (≤ −7 psi), was attached to the chamber and collected throughout the aerosol. Virus concentration in the aerosol was determined by plaque assay titer on the AGI contents (72). Inhaled virus dose was determined as the product of the aerosol concentration, the duration of the exposure (10 min), and the minute volume of the rats. Minute volume was calculated based on weight using Guyton’s formula (73). The dose listed in these studies is the actual presented dose during the aerosol exposure. The presented doses were determined by sampling the air during the aerosol exposure and then performing a plaque assay to calculate the presented dose. Rats were euthanized as they met morbidity criteria, at which point brains and livers were harvested. Tissue titers were obtained following homogenization of tissue and quantified using RT-qPCR or VPA as previously described (30).

### Primary neuron isolation and culture

On the day prior to neuron isolation, acid-washed coverslips were coated with PDL/Laminin (Sigma, P7405-5MG; Invitrogen, 23017-015). Dissociation media (DM) comprising Hanks’ Balanced Salt Solution (Invitrogen, 14175-103) supplemented with 10 mM anhydrous MgCl_2_ (Sigma, M8266), 10 mM HEPES (Sigma, H3375), and 1 mM kynurenic acid were prepared. DM was brought to a pH of 7.2 and sterile filtered prior to use. On the day of isolation, a trypsin solution containing a few crystals of cysteine (Sigma, C7352), 10 mL of DM, 4 µL of 1 N NaOH, and 200 units of Papain (Worthington, LS003126), and a trypsin inhibitor solution containing 25 mL of DM, 0.25 g of trypsin inhibitor (Fisher, NC9931428), and 10 µL of 1 N NaOH were prepared and filter sterilized. Embryonic day 18 Long Evans rats were dissected, and the brains were removed. The cortices were separated from the hippocampus and placed into DM. Five milliliters of trypsin solution was added, and cortices were placed in a 37°C water bath for 4 min, swirling occasionally to mix. The trypsin solution was removed, and cortices were immediately washed with trypsin inhibitor once and then twice more while swirling in the water bath. Following the washes, the trypsin inhibitor was removed and replaced with 5 mL of Neurobasal/B27 media, then triturated to dissociate the neurons. Final volume was brought to 10 mL of Neurobasal/B27, and cells were counted and plated at a density of 50,000 neurons/well for 96-well plates, 100,000–150,000 neurons/well for 24-well plates, or 2 million neurons/well for 6-well plates. One hour after isolation, the media were removed and replaced with fresh Neurobasal/B27 media. Primary neuron cultures were maintained in Neurobasal/B27 media, which consists of standard Neurobasal Plus Medium (Thermo-Fisher, A3582901) supplemented with 1% pen/strep, 1% L-Glut, and 2% B27 Plus Supplement.

### Primary neuron infection

Culture plates (96-, 24- [with cover glass], or 6-well) seeded with primary neurons were transferred into the RBL for infection. WT and attenuated RVFV strains were diluted to an MOI of 0.1 or 3 in DMEM with 2% FBS. At infection, culture media were replaced with media containing viral inoculum and incubated at 37°C with 5% CO_2_ for 1 h with rocking every 15 min. After adsorption, the inoculum was removed and replaced with culture media. The 0 h time point was sampled 15 min after cells were replaced with culture media by diluting supernatant 1:100 in Trizol reagent (Invitrogen, 15596026).

### Cell viability

Neuronal viability was assayed using the LDH-Glo assay (Promega, J2380) as described in the manufacturer’s instructions. Briefly, for the quantification of LDH, supernatant from neurons infected with virus and/or undergoing therapeutic testing was collected and diluted 1:20 in LDH Storage buffer (200 mM Tris-HCl [pH 7.3], 10% Glycerol, and 1% BSA). Samples were stored at −80°C and thawed to room temperature (RT) before use. The LDH Detection Enzyme Mix and Reductase Substrate were thawed to RT and combined immediately prior to use. The LDH positive control (purified LDH from rabbit muscle) was diluted as described. Plates (96-well) were seeded with 50 mL of diluted samples from experiments alongside an eight-point LDH standard. To each well, 50 mL of LDH Detection Reagent was added. Plates were incubated for 60 min at RT. Luminescence was recorded on a BioTek Synergy LX Multi-Mode Reader (Agilent).

### Immunoblotting

Neurons were directly inactivated in radioimmunoprecipitation assay buffer (RIPA) (Thermo Fisher Scientific, 89901) with 1% Halt Protease/Phosphatase Inhibitor (Thermo Fisher Scientific, 78440) for 10 min at room temperature. Tissue homogenate from rats was inactivated by adding 100 mL of homogenate to 900 mL of RIPA buffer with 1% Halt Protease/Phosphatase inhibitor cocktail. Samples were removed from biocontainment and centrifuged at 13,500 relative centrifugal force for 20 min. Cellular debris was removed, and a bicinchoninic acid (BCA) assay was completed following the manufacturer’s instructions (Thermo Fisher Scientific, Pierce BCA Protein Assay, 23227). Fifty micrograms of protein from each sample was loaded into NuPAGE 4%–12% Bis-Tris gel (Invitrogen, NP0323BOX) and run for 45 min at 165 V. The protein was transferred using the iBlot 2 Mini transfer stack (Invitrogen, IB21001) for 7 min onto an Odyssey Nitrocellulose membrane (LICOR Bio, 926-31090). Membranes were blocked for 1 h at room temperature rocking in Intercept (PBS) blocking buffer (LICOR Bio, 927-70001). Following this, membranes were incubated overnight at 4°C rocking with primary antibodies (Table 1) diluted in Intercept T20 (PBS) Antibody diluent (LICOR Bio, 927-75001). The following day, membranes were washed in 1× phosphate-buffered saline (PBS) with 0.1% Tween 20 (PBS-T) three times for 5 min each. Membranes were probed for 1 h at room temperature rocking with IRDye 680RD goat anti-rabbit IgG secondary antibody (LICOR Bio, 926-68071) or IRDye 800CS goat anti-mouse IgG secondary antibody (LICOR Bio, 926-32210). The membranes were washed three times with PBS-T before being imaged on Odyssey CLx (LICOR). Band density was calculated using Image Studio software (LICOR Bio, version 6.0).

**TABLE 1:** Antibodies for immunocytochemistry and immunoblotting

Name of antibody	Company	Source	Catalog no.	Dilution
Cleaved caspase 8	Cell Signal	Rabbit	9429S	1:500
Cleaved caspase 9	Cell Signal	Rabbit	9507S	1:500
Cleaved caspase 3	Cell Signal	Rabbit	9661	1:500
Phospho-RIPK3	Invitrogen	Rabbit	PA5-105701	1:500
Phospho-MLKL	Invitrogen	Rabbit	PA5-105678	1:500
NLRP1	Invitrogen	Rabbit	PA5-116672	1:500
NLRP3	Proteintech	Rabbit	19771-1-AP	1:500
Beta actin	Santa Cruz	Mouse	Sc-47778	1:1,000
RVFV-Gn	BEI	Mouse	NR-43195	1:300
RVFV-N	BEI	Mouse	NR-43188	1:300
RVFV-N	GenScript	Rabbit		1:500
Beta III tubulin	EMD-Millipore	Chicken	AB3954	1:500
Hoescht 33258	Invitrogen	n/a^*a*^	H1398	1:1,000
DRAQ5	Novus Biologicals	n/a	NPB2-81125	1:10,000
Gasdermin D	Cell Signal	Rabbit	39754S	1:500
Gasdermin E	ABCam	Rabbit	ab215191	1:500
RVFV-NSs	Anita McElroy	Rabbit		1:300

^*a*^ n/a, not applicable.

### Immunocytochemistry

Neurons plated on cover glass in 24-well plates were fixed with 4% paraformaldehyde for 15 min at RT. Plates were removed from biocontainment and washed three times in 1× PBS. Cells were permeabilized with 1% Triton-X 100 diluted in PBS for 10 min at RT. Cells were washed three times with 2.5% normal goat serum (Thermo Fisher Scientific, 50062Z) and then blocked with 5% normal goat serum at room temperature for 1 h. Cells were incubated with primary antibodies (Table 1) diluted in 5% normal goat serum for 2 h at RT. Cells were washed with 2.5% normal goat serum three times and then incubated with goat anti-rabbit, goat anti-mouse, or goat anti-chicken secondary antibodies for 1 h at RT. Cells were washed with 1× PBS and incubated with Hoechst stain for 30 s. The Hoescht stain was washed off with 1× PBS, and cover glasses were mounted to slides with gelvatol. Slides were dried overnight before imaging on a Nikon A1 confocal microscope at the Center for Biologic Imaging at the University of Pittsburgh or a Leica DMI8 inverted fluorescent microscope at the Center for Vaccine Research. Images were processed using ImageJ.

### In-cell western

In-cell western assay was adapted from previous studies (74). Briefly, 96-well black/clear bottom plates (ThermoFisher, 165305) were seeded with 50,000 neurons per well. After 4 days in culture, cells were infected at an MOI of 3 of either WT RVFV, RVFV-delNSs/NSm, or mock-infected for 1 h, as described. At 24 hpi, plates were inactivated in 4% paraformaldehyde for 15 min at RT and then washed three times with 200 mL of 1× PBS for 10 min. Plates were blocked with 50 μL of 3% Triton-X100 per milliliter of LiCOR Intercept block (LICOR, 927-70001) for 45 min. Primary rabbit antibodies were diluted 1:500 in antibody solution: one part 1× PBS to three parts LICOR Intercept Block with 3% Triton-X 100 per milliliter. Plates were incubated in 50 μL per well of primary antibody solution for 2 h at RT. Following this, plates were washed three times with 200 μL of 1× PBS for 10 min each. Secondary antibodies were prepared using anti-rabbit 800CW (LICOR, 926-32211, 1:10,000) and DRAQ5 (Novus Biologicals, NPB2-81125, 1:10,000) in antibody solution, and after the final wash, 50 μL was added to each well. Plates were incubated for 1 h at RT in the dark. Three more washes with 1× PBS for 10 min were performed. Plates were imaged on LICOR Odyssey Clx with a focus offset of 4 mm. Signals for 700 and 800 were obtained using the In-Cell Western analysis panel. The signal of the target antibody was normalized to the DRAQ5 signal in each well, and normalized signals were reported as fold change compared to mock.

### Statistical analysis

Statistical analyses were performed using GraphPad Prism software (La Jolla, CA, USA). For Fig. 2E, to compare the signal intensity of protein between uninfected and RVFV-rats, a Student’s *t* test was performed. For Fig. 3A, 4A and B, and 5B through J, a two-way analysis of variance (ANOVA) was performed to compare results from RVFV-infected cells to mock-infected controls. For Fig. 6B and C, a two-way ANOVA was performed to compare drug treatment to untreated (0 μM) controls. For Fig. 6D and E, a two-way ANOVA was performed to compare viral titer with drug treatments to untreated control at 24 and 48 hpi. Multiple comparison was performed using Dunnett’s multiple comparison test. Significance was indicated by *P < 0.05; **P < 0.01; ***P < 0.001; ****P < 0.0001; and ns, no significance.

## Data Availability

All data needed to evaluate the conclusions in the paper are included in the main paper and supplemental material. Reagents are available from the corresponding author upon request.

## SUPPLEMENTAL MATERIAL

Supplemental material jvi.01742-25-s0001.pdf Figures S1 to S3 and Table S1.

ASM does not own the copyrights to Supplemental Material that may be linked to, or accessed through, an article. The authors have granted ASM a non-exclusive, world-wide license to publish the Supplemental Material files. Please contact the corresponding author directly for reuse.
