# Supplementary material for: Rift Valley fever virus activates multiple cell death pathways in neurons
Source: J Virol. 2026 Jan 22;100(2):e01742-25. doi: 10.1128/jvi.01742-25 (PMC12911891; doi:10.1128/jvi.01742-25)
Supplement: Supplemental material — Figures S1 to S3 and Table S1. [file jvi.01742-25-s0001.pdf]

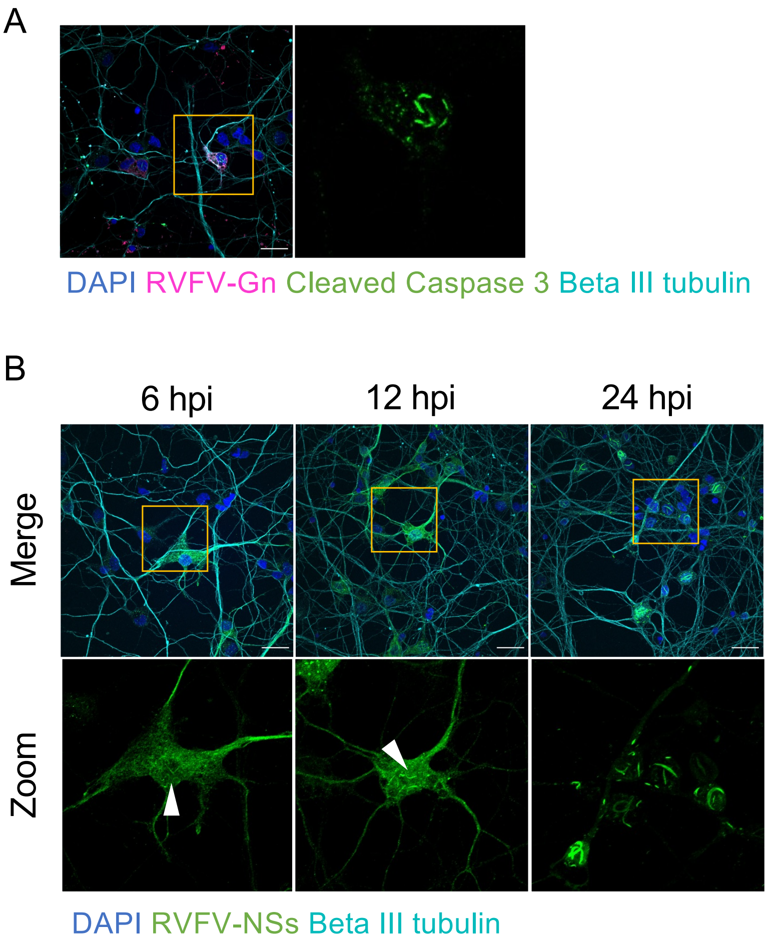

Supplemental Figure 1. RVFV NSs and cleaved caspase 3 filament in the nucleus of RVFV-infected cortical neurons. Primary rat cortical neurons infected at MOI 3 of wildtype RVFV and fixed at 6, 12 and 24 hpi. (A) Neurons fixed at 6 hpi and stained with anti-RVFV-Gn, anti-Cleaved caspase 3, and anti-Beta III tubulin antibodies (left panel), zoom on cleaved caspase 3 (right panel). (C) Wildtype RVFV at 6, 12 and 24 hpi stained with anti-RVFV-NSs and anti-Beta III tubulin antibodies. White arrows indicate NSs filaments at 6 and 12 hpi. Magnification 60X, scale bar = 25 μm.

A

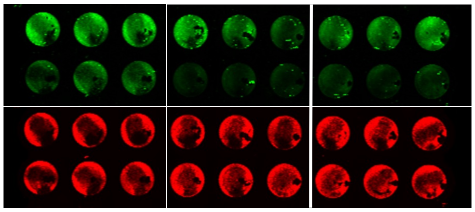

Supplemental Figure 2. Detection of RVFV NSs and NSm by in-cell western. Primary rat cortical neurons infected at MOI 3 of wildtype, RVFV-DelNSs/NSm, or mock-infected and fixed at 24 hpi. (A) Neurons stained with anti-RVFV-NSs antibody (first row) or anti-RVFV-NSm antibody (second row) and counterstained with DRAQ5 (red, third and fourth rows).

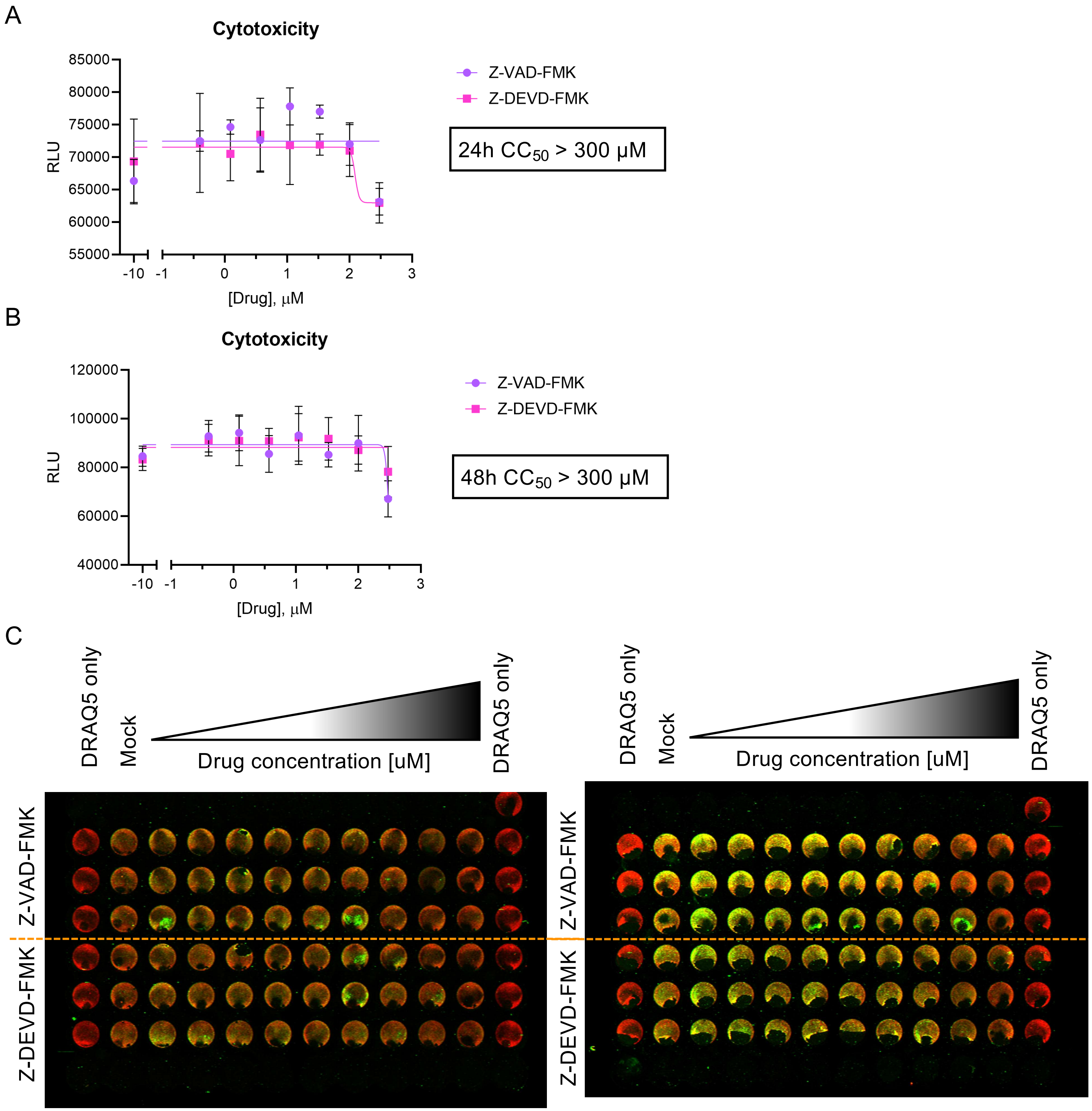

Supplemental Figure 3. 50% cytotoxicity of caspase inhibitors in primary rat neurons and cleaved caspase 3 detection by ICW. Primary neurons were treated with 3-fold dilutions of Z-VAD-FMK or Z-DEVD-FMK for 24 (A) or 48 (B) hours. Toxicity of each was quantitated using CellTiter-Glo (Promega). The 50% cytotoxicity concentration (CC50) was calculated in Graphpad Prism. (C) Plate layout and visualization of cleaved caspase 3 (green) at 24 hours (left) or 48 hours (right) in the presence of increasing concentrations of Z-VAD-FMK or Z-DEVD-FMK. Counterstained with DRAQ5 (red).

**Supplemental Table 1. Assessment of gasdermin antibodies in primary rat cortical neurons**						
Target	Vendor (cat#)	Host	Source	Reactivity	Assay (dilution)	Result
Cleaved Gasdermin D	Cell Signal (36425)	Rabbit IgG	Monoclonal	Human	WB (1:500)	No reactivity
					ICC (1:300)	None
GSDMD	Abcam (ab219800)	Rabbit IgG	Monoclonal	Mouse, Rat	WB (1:500	No bands
GSDMDC1	Santa Cruz (sc-393581)	Mouse IgG	Monoclonal	Mouse, Rat, Human	WB (1:500)	No bands
GSDME/NT	Abcam (ab215191)	Rabbit IgG	Monoclonal	Mouse, Rat, Human	WB (1:500)	55 kDa and 35 kDA bands
					ICC (1:300)	None
GSDMD	Abcam (ab209845)	Rabbit IgG	Monoclonal	Mouse	WB (1:500)	No bands
Cleaved N-terminal GSDMD	Abcam (ab215203)	Rabbit IgG	Monoclonal	Human	WB (1:500)	No bands
Gasdermin D (E9S1X)	Cell Signal (39754S)	Rabbit IgG	Monoclonal	Mouse, Rat, Human	WB (1:500)	No bands
					ICC/ICW (1:500)	Yes
